# A Treatise on Strabismus, with a Description of New Instruments Designed to Improve the Operation for Its Cure, in Simplicity, Ease and Safety; Illustrated by Cases

**Published:** 1842-09-03

**Authors:** 


### BIBLIOGRAPHICAL NOTICE.

*A Treatise on Strabismus, with a description of New Instruments designed to improve the operation for its cure, in simplicity, ease, and safety; illustrated by cases.* By JAMES BOLTON, M. D., A. M., Member of the Medical Society of Virginia. Richmond, 1842. pp. 36.

This little treatise, dedicated to Dr. J. Kearney Rodgers by "his former pupil," contains a concise account of strabismus and its varieties, with a modification of the received operation for its cure. Dr. BOLTON conceives that the instruments which he employs are such as to facilitate materially the operation. His directions regarding the manipulations, and the after treatment, are precise and clear, and strike us as just. Several cases illustrative of the doctor's views and method are appended.

